# Biomarkers in individualized management of chimeric antigen receptor T cell therapy

**DOI:** 10.1186/s40364-020-00190-8

**Published:** 2020-05-11

**Authors:** Mengyi Du, Parameswaran Hari, Yu Hu, Heng Mei

**Affiliations:** 1 Institute of Hematology, Union Hospital, Tongji Medical College, Huazhong University of Science and Technology, Wuhan, 430022 China; 2 Division of Hematology/Oncology, Medical College of Wisconsin (MCW), Milwaukee, WI USA

**Keywords:** CAR-T therapy, Biomarker, Safety, Efficacy, Prognosis

## Abstract

The development of chimeric antigen receptor (CAR) T cell immunotherapy has achieved promising results, both in clinical studies and in commercial products for patients with hematologic malignancies. Despite high remission rates of CAR-T cell therapy in previously untreatable, refractory and/or relapsed patients, several challenges in CAR-T therapy remain to be overcome, especially in integrating such therapies into personalized disease management approaches. Given the unique characteristics of CAR-T therapy, it is particularly urgent to identify biomarkers to maximize their clinical benefits. This systematic review summarizes clinically relevant biomarkers that may help individualized disease management in patients receiving CAR-T cell therapy in terms of toxicity warning, efficacy prediction and relapse monitoring. We summarize data from 18 clinical trials, including traditional indicators like cytokines, biochemical proteins, tumor burden, as well as potential novel indicators such as CAR-T cell expansion and persistency. The establishment of a biomarker-based system aimed at individualized management is recommended to guide better clinical application of CAR-T products.

## Background

With no restriction to major histocompatibility complex (MHC) [1], chimeric antigen receptor (CAR) -T cells have been a breakthrough in personalized cancer therapy, especially in hematological malignancies. Since the development of the first generation 1 of CAR-T cells [2, 3], their structure has been optimized. Currently, fourth generation CAR-T cells are available [4], which provide higher response rates and longer remission duration. The potent anti-tumor effects of CAR-T cells [5, 6] led to accelerated regulatory approval, an extensive investigation of their mechanisms, and the development in clinical investigation of several CAR-T cells targeting different tumor-associated antigens. To date, two anti-CD19 CAR-T products have been approved by the Food and Drug Administration (FDA) for human use, which are known as axicabtagene ciloleucel (axi-cel) and tisagenlecleucel (CTL019) [7]. After the first reports on CAR-T cells from the University of Pennsylvania, an increasing number of institutions around the world have reported the clinical trial of numerous CAR-T products, which are summarized in Table 1. The anti-tumor effects of CD19 targeting CAR-T cells have been extensively explored and reported in patients with B-cell acute lymphocytic leukemia (B-ALL), chronic lymphocytic leukemia (CLL), non-Hodgkin’s lymphoma (non-NHL), and other CD19 positive cancers [8]. The anti-tumor effects of CAR T-cells targeting the B cell maturation antigen (BCMA) have also been investigated in multiple myeloma (MM) [9]. The number of clinical trials involving the use of CAR-T registered on ClinicalTrials.gov (URL: https://clinicaltrials.gov/) is increasing exponentially. Except for CD19 and BCMA, various surface antigens, including CD22, CD20, and CD138, have also been purposed as therapeutic targets in lymphoid tumors [10–12], while CD123, CD33, CD56, and Fms-like tyrosine kinase (FLT3) have been suggested as targets in myeloid neoplasms [13, 14]. The increasing commercial value of CAR T-cell therapies is also reflected in the fact that the number of patents on different CAR T-cell products has increased from less than 100 in 2013 to more than 600 in 2016 [15].

**Table 1:** Influential clinical trials and studies in CAR-T therapy

NO	Institution	NCT	Author	Year	Journal	No.pts	Condition	Target	CD*	Dose	LD*
13	Upeen & CHOP	NCT01626495	Grupp	2013	N Engl J Med	2	B-ALL	CD19	4-1BB	1.4 × 10^6^ - 1.2 × 10^7^/Kg	N/A
			Fitzgerald	2016	Crit Care Med	39	B-ALL				
			Gofshteyn	2018	Ann Neurol	51	ALL				
		NCT02435849 (ELIANA)	Maude	2018	N Engl J Med	75	B-ALL	CD19	4-1BB	0.2 × 10^6^ - 5.4 × 10^6^/Kg	FC*
			Laetsch	2019	Lancet Oncol	58					
		NCT02445248 (JULIET)	Schuster	2019	N Engl J Med	93	DLBCL	CD19	4-1BB	N/A	N/A
			Bishop	2019	Blood Adv	7					
		NCT01029366	Porter	2015	Sci Transl Med	14	CLL	CD19	4-1BB	0.142 × 10^8^–11.3 × 10^8^	Flu*/Cy*/Bendamustine/Pentostatin
		NCT02030834	Schuster	2017	N Engl J Med	28	DLBCL	CD19	4-1BB	1–5 × 10^8^	N/A
		NCT01626495 NCT01029366	Maude	2014	N Engl J Med	51	see in NCT01626495 & NCT01029366				
		NCT02030847** NCT01626495 NCT01029366	Teachey	2016	Cancer Discov	51	ALL	CD19	4-1BB	1.0 × 10^7^ - 5.0 × 10^8^	N/A
		NCT01747486** NCT01029366	van Bruggen	2019	Blood	27	CLL	CD19	4-1BB	1.0 × 10^7^ - 5.0 × 10^8^	N/A
		NCT02640209** NCT01747486 NCT01029366	Fraietta	2018	Nat Med	41	CLL	CD19	4-1BB	1.0 × 10^8^ - 5.0 × 10^8^	N/A
8	FHCRC	NCT01865617	Turtle	2016	J Clin Invest	30	B-ALL	CD19	4-1BB	2 × 10^6^/Kg	FC
				2016	Sci Transl Med	32	NHL				
				2017	J Clin Oncol	24	CLL				
			Gust	2017	Cancer Discov	133	B-ALL & NHL & CLL				
			Hay	2017	Blood	133	B-ALL & NHL & CLL				
				2019	Blood	53	B-ALL				
			Hirayama	2019	Blood	21	NHL				
				2019	Blood	65	NHL				
7	NCI	NCT00924326	Kochenderfer	2017	J Clin Oncol	22	NHL&CLL	CD19	CD28	1.0 × 10^6^–6.0 × 10^6^/kg	FC
				2015	J Clin Oncol	15	NHL&CLL				
			Rossi	2018	Blood	22	NHL				
		NCT01593696	Lee	2015	Lancet	21	ALL&NHL	CD19	CD28	1.0 × 10^6^ or 3.0 × 10^6^/kg	FC
		NCT02215967	Ali	2016	Blood	12	MM	BCMA	CD28	9.0 × 10^6^/kg	FC
			Brudno	2018	J Clin Oncol	16					
		NCT02315612	Fry	2018	Nat Med	15	B-ALL	CD19 & CD22	4-1BB	≥1 × 10^6^/Kg	FC
5	MSKCC	NCT01044069	Brentjens	2013	Sci Transl Med	5	B-ALL	CD19	CD28	1.0 × 10^6^–3.0 × 10^6^/kg	FC/Cy/ Cy + clofarabine
			Davila	2014	Sci Transl Med	16					
			Park	2018	N Engl J Med	53					
				2018	Clin Infect Dis	53					
			Santomasso	2018	Cancer Discov	53					
3	SCRI	NCT02028455	Gardner	2017	Blood	45	B-ALL	CD19	CD28	0.5 × 10^6^–10.0 × 10^6^/kg	Flu/Cy
				2016	Blood	7					
			Finney	2019	J Clin Invest	43					
3	Moffitt Cancer Center	NCT02348216 (ZUMA-1)	Locke	2017	Mol Ther	7	NHL	CD19	CD28	2 × 10^6^/Kg	FC
				2019	Lancet Oncol	108					
	M.D.Anderson Cancer Center		Neelapu	2017	N Engl J Med	101					

*: CD: costimulatory domain; LD: lymphodepletion; Cy: cyclophosphamide; Flu: fludarabine; FC: fludarabine+ cyclophosphamide

**: the NCT trial of CAR-T therapy described in this row

**Fig. 1:**
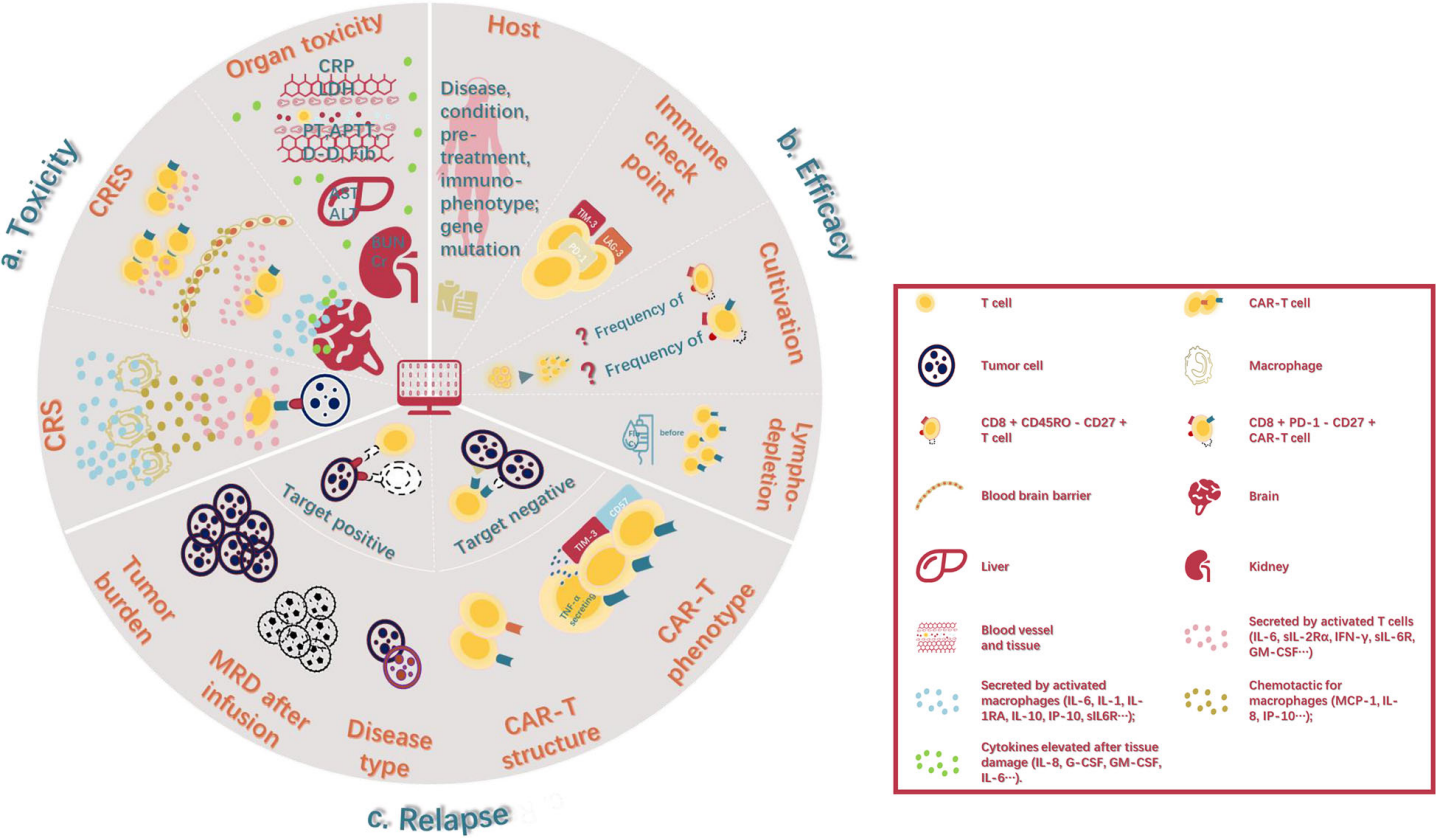
Individualized disease management in terms of toxicity warning ^a^, efficacy prediction ^b^ and relapse monitoring ^c^. **a**. Adverse toxicities in CAR-T therapy are associated with inflammatory cells (e.g., T cells, CAR-T cells, and macrophage), cytokines (e.g., IL-6, IL-10, MCP, GM-CSF, and TNF-γ), and factors related to tissue damage (e.g., CRP, LDH, PT, AST, and Cr). The levels of these factors are useful means of predicting severe toxicity. **b**. Patient characteristics, immune checkpoint expression in T cells before engineering, CAR-T cell cultivation and lymphodepletion are factors affecting the efficacy of CAR-T therapy. **c**. There are two main types of relapse: target-positive and target-negative relapse. To some extent, tumor burden before CAR-T cell infusion, MRD after CAR-T therapy, disease type, structure, and phenotype of CAR-T cells are associated with recurrence. Therefore, they are potential biomarkers of relapse. The precise prediction of toxicity, efficacy, and relapse can contribute to the individualized management of CAR-T cell therapy

Severe cytokine release syndrome (CRS) and CAR-related encephalopathy syndrome (CRES) following CAR-T cell therapy can be life-threatening in some cases [16]. Moreover, it remains unclear to date why some patients exhibit impressive responses to CAR-T cells while others are resistant to such therapies [8]. Disease relapse following CAR-T cell therapy can occur in up to 50% of the patients by 12 months after infusion, and the mechanisms underlying the development of resistance to CAR-T cells remain poorly understood [17]. The lack of robust predictive biomarkers of toxicity and efficacy are significant limiting the individualized management of patients undergoing treatment with CAR-T cells (Fig.1).

A biomarker is defined as “a characteristic that is objectively measured and evaluated as an indicator of normal biological processes, pathogenic processes, or pharmacological responses to therapeutic intervention” (From the National Institutes of Health Biomarkers Definitions Working Group 1998). In the context of CAR-T cell therapies, the “drug” varies between individuals and is capable of self-replication or expansion. Hence, the clinical benefit is heavily dependent on the characteristics of CAR-T cells themselves, including the quantity, function, and persistence prior to engineering, as well as their in vitro and post-infusion in vivo characteristics. These characteristics may serve as powerful predictors of the development of severe side effects, response rates, and duration of response or survival [3, 18, 19], as they determine the properties [20, 21], intensity [22], and duration [23] of CAR-T cell effects. Furthermore, a variety of traditional indices have also been proposed as candidate biomarkers for CAR-T cell therapies. These factors include inflammatory mediators of CRS and CRES [24, 25], markers of organ dysfunction due to severe toxicity [26], and factors associated with the primary disease [27]. In this review, we summarize and discuss various biomarkers that might have clinical value in predicting treatment outcomes and progression-free duration in patients treated with CAR-T cell therapies, thus contributing to the establishment of individualized patient management guidelines.

## Biomarkers predicting toxicity

Safety is the primary endpoint in the majority of phase 1 clinical trials. Based on evidence from several studies [22, 24, 26, 28], CRS and neurotoxicity (CRES) have been suggested to be the most common side effects in patients treated with CAR-T cells. Thus, it is critical to identify biomarkers predicting the development of CRS and CRES.

### Cytokine release syndrome (CRS)

The most common toxicity associated with CAR-T cell therapy is CRS, which is a form of systemic inflammatory response characterized by non-infectious fever, hypotension, hypoxia, and/or multiorgan toxicity. CRS usually develops in the first or second week after CAR-T infusion [29]. However, the severity of CRS symptoms can vary significantly, ranging from low grade with mild symptoms to high grade with early-onset high fevers, hyperpyrexia [26], high incidence of infections [30], and neurotoxicity [26]. Moreover, severe CRS can be accompanied by excessive macrophage activation, coagulation dysfunction, tumor lysis syndrome, and life-threatening multiorgan dysfunction [22–24, 31]. In terms of management, mild to moderate CRS is usually self-limited and can be controlled with close observation, hydration, and supportive care. In contrast, severe CRS requires intensive medical management with tocilizumab alone or in combination with steroids [31]. The management of CRS depends heavily on the risk grade system [32, 33]. Although the grading system takes into account various criteria [34], it is mainly based on clinical signs and subjective symptoms, which is not ideal for grading CRS, as its manifestation can vary immensely.

CRS is triggered by the activation of T cells after engagement of their CARs with cognate antigens expressed on the surface of tumor cells [35]. The activated T cells release various cytokines and chemokines, including interleukin (IL)-6, interferon (IFN)-γ, granulocyte-macrophage colony-stimulating factor (GM-CSF), and soluble IL-2Rα [24, 36]. These cytokines activate monocytes, macrophages and other immune cells, which, in return, release inflammatory cytokines, such as IL-1, IL-6, IL-10, IL-1RA, IFN-γ, and interferon γ-induced protein (IP)-10 [22, 24, 37, 38] promoting tissue damage and multiorgan dysfunction [24]. The interaction between immune cells, tumor cells, cytokines, and necrotic tissue accelerate the pathophysiology of CRS [16]. Hence, CRS is determined by a complex network that includes cells (tumor cells, activated T cells or CAR -T cells, monocytes, and macrophages), inflammatory mediators, and other mediators released by damaged tissues or organs.

Based on the pathologic mechanisms underlying CRS, several groups of indicators have been recommended to monitor CRS. Inflammatory indicators, including cytokines, ferritin, and C-reactive protein (CRP), are the most commonly used predictive biomarkers [39]. The expression profile of cytokines have been investigated in B-ALL [22, 24], CLL [40], and diffuse large B-cell lymphoma (DLBCL) patients [25] treated with anti-CD19 CAR-T cell therapy, in an effort to identify predictive factors for CRS [24]. Peak levels of 24 cytokines were increased in patients and associated with severe CRS, while other 19 cytokines were of no statistically difference, such as IL-2 [24]. The cytokines associated with severe CRS could be divided into four categories: T cell-activating cytokines (e.g., IL-6, IFN-γ, sIL-2Rα, sIL-6R, and GM-CSF), monocyte/macrophage-activating cytokines (e.g., IL-6, IL-1Ra, IL-10, IP-10, and sIL-6R), monocyte/macrophage-attracting chemokines (e.g., monocyte chemoattractant protein (MCP)-1, IP-10, and IL-8), and cytokines released after tissue damage (e.g., IL-6, IL-8, granulocyte Colony-Stimulating Factor (G-CSF, and GM-CSF) [24, 40, 41]. Besides these cytokines, a positive association between the levels of CRP or ferritin and severe CRS was reported [23, 24, 40–42]. IL-6, CRP, ferritin, IL-10, IL-15, and MCP-1 have been investigated as predictive factors for CRS in large populations (Table 2). The predictive value of IFN-γ, IL-15, and GM-CSF has been inconclusive, likely due to differences in study design, patient populations, disease type, or the CAR-T cell platform used.

**Table 2:** Various biomarkers in CAR-T therapy

Biomarker	Toxicity		Re-sponse	Re-lapse	Reference	Biomarker	Toxicity		Re-sponse	Re-lapse	Reference
	sCRS	sCRES					sCRS	sCRES			
Inflammatory:cytokine & CRP & ferritin						Intrinsic CAR-T Cell					
IL-6	+	+	/	/	Maude [23]; Porter [40]; Lee [41]; Teachey [24]; Turtle [20]; Neelapu [25]; Gust [28]; Santomasso [22]	peak CAR-T cell expansion	−/+	+	+	+	Neelapu [25]/ Lee [41]; Porter [40]; Turtle [20]; Kochenderfer [11]; Santomasso [22]; Park [43]; Locke [44]
CRP	+	+	/	/	Maude [23]; Davila [42]; Porter [40]; Lee [41]; Teachey [24]; Fitzgerald [45]; Turtle [20]; Gust [28]; Santomasso [22]; Karschnia [46]	CD8 (+) CD45RO (−) CD27 (+) before engineering	/	/	+	/	Fraietta [21]
Ferritin	−/+	+	/	/	Neelapu [25]/ Maude [23]; Porter [40]; Teachey [24]; Fitzgerald [45]; Turtle [20]; Gust [28]; Santomasso [22]; Karschnia [45]	CD27 (+) PD-1 (−) CD8 (+)	/	/	+	/	Fraietta [21]
IFN-γ	−/+	+	/	/	Porter [40]/ Maude [23]; Lee [41]; Teachey [24]; Turtle [20]; Gust [28]; Santomasso [22]	TNF-α(+) CD8(+)	/	/	/	+	Finney [47]
IL-10	+	+	/	/	Teachey [24]; Turtle [20]; Neelapu [25]; Kochenderfer [11]; Santomasso [22]	TIM-3(+) CD8(+)	/	/	/	+	Finney [47]
IL-15	−/+	+	+	/	Teachey [24]/ Turtle [20]; Neelapu [25]; Kochenderfer [11]; Santomasso [22]; Gofshteyn [48]	CD8(+)	+	/	/	/	Maude [23]
IL-2	–	+	/	/	Porter [40]; Teachey [24]; Turtle [20]; Neelapu [25]; Santomasso [22]; Gofshteyn [48]	CD3(+)	+	/	/	/	Maude [23]
MCP-1	+	+	/	+	Teachey [24]; Hay [26]; Santomasso [22]; Hirayama [49]	Tissue damag					
GM-CSF	−/+	+	/	/	Neelapu [25]/ Teachey [24]; Santomasso [22]	Fibrinogen	+	+	/	/	Teachey [24]; Fitzgerald [45]; Santomasso [22]
TNF-α	+	+	/	/	Teachey [24]; Gust [28]	vwF	+	/	/	/	Hay [26]
IL-8	+	+	/	/	Teachey [24]; Turtle [20]; Santomasso [22]	Ang 2	+	+	/	/	Hay [26]; Santomasso [22]
sIL-2R	+	/	/	/	Maude [23]; Porter [40]; Teachey [24]	D-Dimer	+	+	/	/	Santomasso [22]; Hay [26]
G-CSF	+	+	/	/	Teachey [24]; Santomasso [22]	PT	+	+	/	/	Teachey [24]; Hay [26]; Santomasso [22]
IL-1R	+	+	/	/	Teachey [24]; Santomasso [22]	APTT	+	+	/	/	Teachey [24]; Santomasso [22]; Hay [50]
IP-10	+	+	/	/	Teachey [24]; Santomasso [22]	INR	+	/	/	/	Fitzgerald [45]
IL-1	+	+	/	/	Teachey [24]; Santomasso [22]	PLT	+	+	/	/	Fitzgerald [45]; Santomasso [22]
sIL-6R	+	/	/	/	Teachey [24];	LDH	+	/	/	+	Teachey [24]; Fitzgerald [45]; Hay [50]; Karschnia [46]
IL-7	/	/	/	+	Hirayama [49]	AST	+	/	/	/	Teachey [24]
Primary disease						ALT	−/+	/	/	/	Teachey [24]/Fitzgerald [45]
disease burden	+	+	–	+	Brentjens [51]; Maude [23]; Lee [41]; Turtle [20]; Santomasso [22]	BUN	+	/	/	/	Teachey [24]
immune check-point	/	/	−/+	/	Schuster [18]/ Schuster [52]	Cr	+	/	/	/	Teachey [24]; Fitzgerald [45]

+:There is a statistical difference;

-: There is no statistical difference;

−/+: controversial, and negative references are set before “/”, positive references are set after “/”;

/; not mentioned

The cytokine IL-6 is produced by a variety of cells and can promote T cell proliferation and B cell differentiation, as well as inhibit apoptosis and stimulate the production of the acute-phase proteins CRP and ferritin [53, 54]. The levels of the chemotactic factors IL-8, MCP-1, and IP-10 may indicate mononuclear phagocyte activation in response to systemic inflammation and endothelial damage [24]. IFN-γ, G-CSF, and GM-CSF have been suggested as essential players in endothelial cell injury, which frequently occurs during inflammation-associated tissue damage [55]. On the other hand, IL-10 inhibits the ability of macrophages to stimulate cytokine secretion by T helper type 1 (Th1) cells [56], increasing the cytokine storm as a response. Two recent pre-clinical studies [37, 38] indicated that IL-6, IL-1, and macrophage-derived cytokines were critical factors determining CRS severity, while T cell-derived cytokines were less important. IL-1 might be a key player in the development of CRS, and it promotes the secretion of IL-6 and sIL-6R. These two studies also suggested that the cytokine profile associated with hemophagocytic lymph-histiocytosis/macrophage-activation syndrome (HLH/MAS) mirrors the cytokine profile observed in severe CRS [24] and that IL-2, which is released only by T cells, does not associate with CRS severity [57].

Except for inflammatory factors, immune cells and tumor cells also play vital roles in the cytokine storm network. Notably, in biomarker discovery approaches, the fact that CAR-T are cell products rather than chemical compounds needs to be taken into consideration. CAR-T cells, upon binding to the appropriate antigen, they initiate a cascade of inflammatory reactions in vivo, hence intrinsic characteristic of CAR-T cells, such as the levels of CD3 and CD8 expression, could serve as potential biomarkers (Table 2) [28, 58–60]. The relationship between the peak levels of CAR T-cells and CRS severity remains controversial, likely emanating from the fact that macrophages and not activated T cells seem to be the key mediators of CRS [37, 38]. The predictive value of the characteristics of immune cells has been more extensively explored in efficacy than toxicity. Potent tumor cell elimination and high response rates may be coupled with a higher prevalence of severe CRS. As with tumor cells, disease burden is detected by cytology of bone marrow blasts or minimal residual disease (MRD). Interestingly, higher disease burden before enrollment [41] or at baseline [23] has been linked to a high risk of toxicity.

Coagulopathy, tumor lysis syndrome (TLS), HLH/MAS, and organ dysfunction can develop during CRS [31], especially in patients with severe CRS. Except for HLH/MAS, which has similar pathology to CRS [24, 59], other symptoms or complications may develop due to tissue or organ damage, which correlates with the severity of CRS. Hay et al. demonstrated that the levels of angiopoietin-2 (Ang2) and von Willebrand factor (vwF) were increased in patients with severe CRS and that they could be used as predictive biomarkers. Ang2 and vwF are released from the Weibel-Palade bodies upon endothelial activation, promoting capillary leak [61] and initiation of coagulation. Even though the mechanisms that lead to endothelial activation in CRS have not been fully characterized, the cytokines IL-6 and IFN-γ seem to be involved [26]. Common indicators of abnormal coagulation, including prolonged prothrombin time (PT), activated partial thromboplastin time (APTT), elevated D-dimer, and hypofibrinogenemia, have been reported in grade ≥ 4 CRS [26, 62]. Lactate dehydrogenase (LDH) is a clinical biomarker of TLS [63, 64], and TLS is directly linked to disease burden [65]. This might explain why the peak levels of LDH associate with high-grade CRS [24] and why the median LDH level in grade 3 and 4 CRS patients associates with ferritin levels [45]. Besides, indicators of impaired liver and kidney function, such as AST and BUN, can be used as biomarkers of severe CRS [24, 45].

### CAR-related encephalopathy syndrome (CRES)

CRES is another prominent toxicity occurring in 20–64% of patients undergoing CAR T-cell therapy, with a median onset of 4–5 days after CAR-T cell infusion; in some cases, CRES can be concurrent with CRS [28]. The most common symptoms of CRES include encephalopathy, headache, delirium, anxiety, tremor, aphasia, and other neurotoxicity-related symptoms. Severe CRES (sCRES) emerges with earlier onset and is characterized by a longer duration and shorter progression-free survival (PFS) compared with mild CRES [46]. Though patients with high-grade CRS are more likely to develop high-grade CRES, fatal neurotoxicity may occur even in patients with mild CRS [22, 46]. The treatment of CRES depends on the severity, which is defined according to the Common Terminology Criteria for Adverse Events (CTCAE) criteria [31, 34]. CRES management includes supportive care, or aggressive ICU-level care with anti-epileptics, high-dose corticosteroids, and medications for status epilepticus and cerebral edema. Existing indicators alone are insufficient to predict CRES severity.

Thus far, the exact mechanisms of CAR-T cell-associated neurotoxicity remain poorly understood. Some studies have suggested direct toxicity by CAR-T cells and indirect toxicity caused by the increased levels of inflammatory cytokines in cerebrospinal fluid (CSF), which lead to endothelial cell activation and increased blood-brain barrier (BBB) permeability [28]. Several studies have suggested the presence of CAR-T cells in the central nervous system (CNS), yet it remains unclear why CAR-T cells migrate to the CNS in the absence of intracranial tumors [23, 41, 42, 66, 67]. Among all cytokines, IL-1 and IL-6 have been described as the key players of CRES [38]. More importantly, IL-1 precedes IL-6 secretion by many hours and is capable of inducing the secretion of IL-6 in circulating monocytes. Interestingly, anakinra, an IL-1 receptor antagonist, provided higher clinical benefit than tocilizumab in a murine model [38]. Based on currently available evidence, the pathological processes of CRES are mediated by inflammatory factors, CAR-T cells, and endothelial cells.

Several similarities between CRS and neurotoxicity have been described, including high cytokine concentrations in the serum, high CAR-T cell counts in the blood, and dysfunction of tissues and organs. Similarly to CRS, higher levels of inflammatory mediators, including IL-1, IL-6, IL-10, IL-2, IFN-γ, tumor necrosis factor (TNF)-α, GM-CSF, G-CSF, MCP-1, CRP, and ferritin, are associate with higher grade neurotoxicity [22, 25, 28, 37, 38, 40, 46, 68]. Santomasso et at. have indicated shown that patients with severe neurotoxicity had upregulated higher levels of IL-1 and IL-6 by day three after CAR-T cell infusion, suggesting that increased levels of these cytokines early after treatment can predict the early rise and higher peak of them were associated with severe CRES [22]. Despite the potent effects of IL-1 shown in pre-clinical studies [37, 38], seldom clinical studies have revealed its role in neurotoxicity in detail. Therefore, the role of IL-1 in immune responses and its potential use as a target to treat human diseases merits further investigation. The peak levels of MCP-1, IP-10, and IL-8 in CSF were more distinct higher than in the serum when compared with baseline level [22]. Elevated concentrations of MCP-1, IP-10, and IL-8 might be indicative of activated microglia, macrophages, or astrocytes in response to systemic inflammation and endothelial damage. Earlier elevation and higher peak levels of CAR-T cells in the blood have been associated with severe CRES [22, 28]. Among the factors associated with CAR-T cell expansion, such as disease burden prior to infusion, the phenotype of CAR-T cells before and after genetic engineering, and the dose of cell infusion, it remains unclear which factors play decisive roles in CAR-T peak levels in vivo. Endothelial cell dysfunction is consistent with clinical evidence of coagulatory abnormalities and disseminated intravascular coagulation (DIC) in patients with severe encephalic toxicity. Gust et al. [28] demonstrated that severe neurotoxicity was accompanied by DIC, with elevated prothrombin time, activated partial thromboplastin time, and d-dimer as early as 2–5 days after CAR-T cell infusion. Moreover, they found that prolonged thrombocytopenia and a late reduction in fibrinogen to a nadir approximately one to two weeks after CAR-T cell infusion were associated with the development of severe neurotoxicity.

The main difference between CRS and CRES is the IL-2 level, which has been associated with severe CRES [22, 25, 48, 68], but not severe CRS. IL-2 stimulates the proliferation and maturation of intracranial cells that are derived from mononuclear macrophages, such as oligodendrocytes and astrocytes [57]. However, whether and to what extent this effect contributes to the development of CRES remains unclear. This discrepancy provides scientists a window to probe into the unique mechanisms underlying the pathogenesis of CRES.

### Early prediction

With plenty of candidates to choose from, the serum levels of IL-6 and IFN-γ in the first 24 h after CAR-T cell infusion in B-ALL patients have been reported as robust biomarkers of severe CRS and CRES by scientists from the Fred Hutchinson Cancer Research Center (FHCRC) [26, 68]. In NHL patients, high serum levels of IL-8, IL-10, and IL-15, as well as low levels of transforming growth factor (TGF)-β could also predict the development of severe CRS and neurotoxicity [68]. Because IL-8, IL-10, IL-15, and TGF-β concentrations were not assessed in the B-ALL cohort of the study NCT01865617, it remains unknown whether these cytokines predict toxicity in B-ALL. Identification of biomarkers on day one after CAR-T cell infusion provides an opportunity to test whether early intervention strategies in high-risk patients may mitigate or prevent severe toxicity and whether early prevention of severe toxicity will impact the efficacy of CAR-T cells. A combination of robust predictive biomarkers at an early stage needs to be integrated into a precise and sensitive risk grading system to guide therapy decisions.

## Biomarkers predicting efficacy

Factors that can affect the efficacy of CAR-T cell therapy vary, and include the patient and disease characteristics, CAR-T cell culture-related procedures, and lymphodepletion prior to T cell infusion.

### Patient evaluation

Individual traditional factors, such as patient age, prior therapy, peripheral tumor burden, p53 status [18, 21, 25], the presence of chromosome 17p deletions, and immunoglobulin heavy chain variable region gene IGH variable (IGHV) mutation status [40], are not associated with response to CAR-T cell therapy. Assessment of the expression of programmed death-1 (PD-1), lymphocyte activation gene-3 (LAG-3), and T cell immunoglobulin-3 (TIM-3) on tumor cells and their receptors on immune cells by immunohistochemical analysis, revealed that high expression of these molecules is associated with a lower response to CTL019 therapy [52]. However, a following study by the same scientists showed contradicting results, with no differences in response based on tumor expression of CD19 or immune checkpoint-related proteins [18]. Therefore, we believe that patients should not be excluded from CAR-T therapy based on age, prior lines of therapy, tumor burden, presence of mutations or expression levels of immune checkpoint molecules, though these characteristics should be taken into account for patient enrollment in clinical trials.

### CAR -T cell cultivation

Several studies, including a phase 2 clinical trial of ZUMA-1, suggested that higher CAR-T- cell levels in the blood are associated with response [25, 44]. Similar results were found in the NCT00924326 study [19] conducted by the National Cancer Institute (NCI) and NCT01029366 conducted by UPenn [40]. Fraietta et al. [21] performed genomic, phenotypic, and functional analyses in 41 CLL patients who were enrolled in clinical trials assessing the use of CTL019 therapy. They identified that CD8^+^CD45RO^−^CD27^+^ T cells in leukapheresis samples were resting, long-lived, memory lymphocytes, which could rapidly expand and acquire effector functions after antigen re-exposure. They also reported that the frequency of these cells was associated with the response rate. They also indicated that the population of CD27^+^PD-1^−^CD8^+^ CAR-T cells expressing high levels of the IL-6 receptor might be associated with response and tumor control. Rossi et al. showed that the pre-infusion of polyfunctional CAR-T cells was significantly associated with clinical response to CAR-T cell therapy [69]. These findings underscore the potential of using biomarkers predicting response in guiding the patient selection and optimizing CAR-T-cell production prior to infusion.

### Lymphodepletion

A recent meta-analysis [70] using data from 320 patients from 14 studies to estimate the response to autologous CD19 CAR-T cell therapy suggested that patients who received lymphodepletion with cyclophosphamide (Cy) or fludarabine (Flu) showed a better response (77%; 95% CI, 67–83%; *P* = 0.001) compared with non-lymphodepleted patients (66%; 95% CI, 41–83%). Furthermore, they found that the administration of higher doses of lymphodepleting agents was associated with higher MCP-1 and IL-7 concentrations after T cell infusion [49], and high MCP-1 and IL-7 levels have been associated with good prognosis. Therefore, whether the extent of lymphodepletion can affect the efficacy of CAR T-cell therapies requires further investigation. Additionally, the optimal duration of lymphodepletion, as well as its combination with other chemotherapies or radiotherapy remain to be established.

## Prognostic biomarkers of relapse

Relapse can occur in up to 50% of B-ALL patients within 12 months after CAR-T cell infusion. The interaction between the antigen receptor and target plays a decisive role in the efficacy of CAR-T cell therapy; hence, loss or dysfunction of either one may contribute to disease relapse [27, 71]. Early relapse has been reported in antigen-positive patients, while late relapse is typically associated with antigen loss [50]. Antigen-positive relapse usually occurs within the first few months after successful induction of remission and is often associated with limited CAR-T cell persistence and transient B cell aplasia, suggesting a loss of active CAR-T cell-mediated surveillance [23]. Target antigen loss is a well-demonstrated mechanism of disease relapse following successful remission in patients treated with CAR-T cells, yet the mechanisms underlying antigen loss need further investigation.

Scientists from the Seattle Children’s Research Institute confirmed that the expansion of CAR-T cells is less robust in patients with short B cell aplasia (BCA) than in patients with long BCA [47]. They found that the primary driver of CAR-T cell expansion and that minimizes the risk of CD19^+^ relapse was the cumulative burden of CD19-expressing cells, as assessed in the bone marrow prior to lymphodepleting chemotherapy. In contrast to other reports, neither the cell dose [27] nor leukemia burden [43, 72] alone was a predictor of the magnitude or duration of CD19 CAR-T engraftment in this trial. The authors also suggested that a high antigen burden does not induce exhaustion of CAR T-cellsthe therapeutic cells, and that the elimination of the target cells promotes the transition of the effector cells into functional memory CAR-T cells. As with CAR T cell persistency, there are important differences between the T cell products. For instance, T cells with 4-1BB co-stimulatory CARs tend to persist longer than T cells with CD28 co-stimulatory CARs. 4-1BB-based CAR-T cells persisted in the blood for a median duration of 168 days (range 20–617 days) in patients B cell aplasia who remained in remission, while CD28-based CAR-T cell persistence has been the median duration of 30 days, and these cells are rarely detected beyond 3 months [17]. Phenotypic and functional attributes of CAR-T cells are associated with duration. Finney et al. [47] analyzed the phenotype and function of subtypes in relation to the remission duration. In patients with long-term remission, the percentage of CD8^+^ CAR-T cells secreting TNF-α was higher, while the percentage of CD8^+^ CAR-T cells expressing TIM-3 was significantly lower. Except for the phenotypes with prognostic value, the frequency of CD8^+^ CAR-T cells secreting IFN-γ or IL-2, as well as the frequency of CD8^+^ CAR T-cells expressing PD-1 or LAG-3 did not show significant differences.

The characteristics of the primary disease also have prognostic value. Disease burden detected by the cytological assessment of bone marrow or MRD can predict, to some extent, remission duration. Disease histology can also predict relapse. In a CTL019 lymphoma cohort, the PFS of patients with follicular lymphoma was found to be longer compared to patients with DLBCL [52]. A lower pre-lymphodepletion LDH level (hazard ratio (HR), 1.39; 95% CI, 1.12–1.74 per 100 U/L increment, *P* = 0.003) or a higher pre-lymphodepletion platelet count (HR, 0.65; 95% CI, 0.47–0.88 per 50,000/μL increment increase, *P* = 0.006) independently associated with better event-free survival (EFS) [50].

Upon interaction between CAR-T cells and tumor cells, complex downstream signaling cascades promote immune cell-mediated cancer cell killing. In the acute setting, biomarkers, such as elevation of cytokines and elimination of certain tumor cell clones, can help identify patients with long-term survival. NHL patients with higher baseline MCP-1 levels or peak IL-7 levels after T cell infusion have a lower incidence of disease progression [49]. Complete remission (CR) after T cell infusion indicates a longer PFS regardless of the disease type [49, 73] and institution where the study has been conducted [18, 44]. Among CR patients, high-throughput sequencing (HTS) before and after CAR T-cell infusion revealed that the elimination of leukemic cell clones was associated with improved EFS (median event-free survival of 8.4 versus 3.6 months, *P* = 0.036) [50].

In patients determined as high-risk of relapse after CAR-T therapy, consolidation therapy after infusion will be imperative to prolong survival. A study conducted in our institute highlighted that CAR-T therapy following allogeneic hematopoietic stem cell transplantation (allo-HSCT) was a safe and effective therapeutic strategy for relapsed/refractory B-ALL patients, and might prolong EFS and RFS, especially in patients with high pre-infusion tumor burden [72]. Hence, the identification of biomarkers is crucial for early identification, accurate intervention, and individualized management.

## Challenges and future perspectives

A major challenge in the development of cancer immunotherapy biomarkers will be the integration of comprehensive bioinformatics data to traditional clinical symptoms, as well as the delineation of the intrinsic mechanisms linking biomarkers to clinical outcomes or phenotypes. Besides biomarkers predicting toxicity, efficacy, and patient survival, extensive efforts to identify novel antigen targets have been made over the last decade, yet with limited breakthrough. The challenge of antigen-receptor design extends to the solid tumor field looking for unique tumor-specific surface antigens and the elucidation of the role of the immunosuppressive tumor microenvironment (TME) [74–76]. Combination therapies provide encouraging clinical benefits in patients with hematologic and solid malignancies. T cells contain two complete and independent CARs, have overcome, to some extent, the impact of antigen escape in lymphoma [77] and myeloma [78] patients. Novel high-throughput technologies, such as single-cell RNA-sequence, for biomarker identification [69], combination therapy with PD-1 inhibitors, and the utilization of universal T cells may offer virtually unlimited potential for cancer immunotherapy and maximize clinical benefit in cancer patients [79].

## Conclusion

Biomarkers play an important role in personalized management in terms of toxicity, efficacy prediction, and relapse assessment. The discovery of new biomarkers and validation of existing ones should be of high importance so that they can be incorporated into routine clinical practice. The combination of modified CAR-T products and individualized personalized management is imperative to maximize the clinical benefits of CAR-T cell therapy and expand the availability of this promising therapy in a broader range of patients.

## Data Availability

The material supporting the conclusion of this review has been included within the article.

## Abbreviations

APTT: Activated partial thromboplastin time
HLH/MAS: Hemophagocytic lymph-histiocytosis/macrophage-activation syndrome
Ang2: Angiopoietin-2
HTS: High-throughput sequencing
axi-cel: Axicabtagene ciloleucel
IFN: Interferon
B-ALL: B-cell acute lymphocytic leukemia
IGHV: IGH variable
BBB: Blood-brain barrier
IL: Interleukin
BCA: B cell aplasia
IP: Interferon γ induced protein
BCMA: B-cell maturation antigen
LDH: Lactate dehydrogenase
CAR: Chimeric antigen receptor
MCP: Monocyte chemoattractant protein
CLL: Chronic lymphocytic leukemia
MHC: Major histocompatibility complex
CR: Complete remission
MM: Multiple myeloma
CRES: CAR-related encephalopathy syndrome
MRD: Minimal residual disease
CRP: C-reactive protein
MSKCC: Memorial Sloan Kettering Cancer Center
CRS: Cytokine release syndrome
NCI: National Cancer Institute
CSF: Cerebrospinal fluid
non-NHL: Non- Hodgkin's lymphoma
CTCAE: Common Terminology Criteria for Adverse Events
PFS: Progress-free survival
CTL019: Tisagenlecleucel
PT: Prolonged prothrombin time
Cy: Cyclophosphamide
R/R: Relapse/refractory
DIC: Disseminated intravascular coagulation
sCRES: Severe CRES
DLBCL: Diffuse large B-cell lymphomas
CRS: Severe CRS
EFS: Event-free survival
TGF: Transforming growth factor
FDA: Food and Drug Administration
TLS: Tumor lysis syndrome
FHCRC: Fred Hutchinson Cancer Research Center
TME: Tumor microenvironment
FLT3: Fms-like tyrosine kinase
TNF: Tumor necrosis factor
Flu: Fludarabine
UPenn: University of Pennsylvania
G-CSF: Granulocyte colony-stimulating Factor
vwF: Von Willebrand factor
